# Buffer therapy in metabolic acidosis after surgery-associated hemorrhage in pediatric oncology

**DOI:** 10.1186/cc10754

**Published:** 2012-03-20

**Authors:** N Matinyan, A Saltanov, I Letyagin, O Obukhova

**Affiliations:** 1 N.N. Blokhin Russian Cancer Research Center, Moscow, Russia

## Introduction

Surgery in pediatric oncology is usually massive and traumatic and often leads to acute blood loss, which can result in metabolic acidosis. To treat acidosis, sodium bicarbonate is often used; however, its application has some side effects. In this situation *tris*-hydroxymethyl aminomethane (THAM) seems to be more effective. The objective of this study was to evaluate the effect of THAM for treating metabolic acidosis after surgery-associated hemorrhage in pediatric oncology.

## Methods

The observational study included 50 children aged 12 months to 16 years (among them 27 boys) with metabolic acidosis after surgery-associated hemorrhage: 40% patients lost 58 ± 8.5% of total blood volume, 26% lost 150 ± 9.5% of total blood volume. Patients received 3.66% THAM infusion. The dose of THAM infusion was calculated as the dose administered (ml):negative standard BE (mmol/l)×kg body weight, and did not increase 1.5 ml/kg body weight every 24 hours. The following were analyzed: Na^+^, K^+^, ionized calcium, lactate, pH, pCO_2_, HCO_3 _and BE of arterial blood, before therapy, and after receiving a one-half dose and a full dose of THAM. The significance of differences was assessed by Student's *t *test, Mann-Whitney coefficient and chi-square test; *P *< 0.05 was considered statistically valid.

## Results

There were no differences in the concentrations of electrolytes and lactate. At the stages of the research the following significant dynamics have been noted: pH (7.27 ± 0.01; 7.31 ± 0.01; 7.35 ± 0.01; *P *< 0,01), HCO_3 _(18.59 ± 0.26; 19.5 ± 0.3; 21.2 ± 0.41 mmol/l; *P *< 0.01) and BE (-8.34 ± 0.3; -6.58 ± 0.37; -4.47 ± 0.45 mmol/l; *P *< 0.01). PaCO_2 _tension did not change significantly (38.9 ± 0.83; 37.3 ± 0.94; 37.5 ± 0.95 mmHg; *P *>0.05).

## Conclusion

THAM infusion resulted in metabolic acidosis correction without the development of hypernatremia and increase of CO_2 _tension. However, the small number of observations does not allow one to assess accurately the clinical effect of THAM for these patients.

